# Post-exposure booster vaccination recalls vaccine-induced memory and accelerates *Bordetella pertussis* clearance in murine lungs and trachea

**DOI:** 10.1038/s41598-026-56963-y

**Published:** 2026-07-20

**Authors:** Marie Ballester, Paola Fontannaz, Floriane Auderset, Renato Gualtieri, Paul-Henri Lambert, Claire-Anne Siegrist

**Affiliations:** 1 Division of Pediatrics, Department of Women-Mother-Child, Lausanne University Hospital and University of Lausanne, rue du Bugnon 50, 1011 Lausanne, Switzerland; 2 Center for Vaccine Immunology, Departments of Pathology- Immunology and Pediatrics, University of Geneva, Geneva, Switzerland; 3 Department of Pediatrics, Gynaecology and Obstetrics, Faculty of Medicine, University of Geneva, Geneva, Switzerland

**Keywords:** Vaccine, Pertussis, Memory, Antibody, Carriage, Clearance, Colonization, Diseases, Immunology, Microbiology

## Abstract

Despite high vaccination coverage, pertussis continues to cause substantial morbidity in infants, largely because acellular pertussis (aP) vaccines do not prevent bacterial carriage and transmission. Whether post-exposure booster vaccination can rapidly reduce respiratory carriage and thereby limit transmission remains unclear. We used an adoptive murine transfer model mimicking waning vaccine-induced antibodies observed in humans to assess the impact of post-exposure aP vaccination following respiratory challenge with *Bordetella pertussis*. Bacterial loads in the lungs and trachea and pertussis-specific antibody responses were assessed across multiple independent experiments. Post-exposure aP vaccination triggered a rapid recall of pertussis-specific antibodies and significantly accelerated bacterial clearance from both lungs and trachea. Compared with non-boosted immune mice, vaccinated animals showed significantly lower bacterial loads on days 7, 10, and 14 after exposure and cleared *Bordetella pertussis* earlier (*p* < 0.01). Similar reductions in bacterial burden were observed with delayed boosting, lower-antigen-content vaccines, and passive immunization. In this preclinical model, post-exposure pertussis booster vaccination efficiently recalls memory B cells and accelerates *Bordetella pertussis* clearance in the lung and upper airways. These findings support the potential role of post-exposure vaccination strategies as a complementary approach to limit transmission in household and community settings and thus better control epidemics.

**Supplementary Information:**

The online version contains supplementary material available at 10.1038/s41598-026-56963-y.

## Introduction

Despite paediatric vaccination coverage of roughly 85% globally, *Bordetella pertussis* (*B. pertussis*) is still causing significant morbidity and mortality worldwide, and its prevalence is increasing in countries where acellular pertussis (aP) vaccines are preferred^1–4^. Since 1996, aP vaccines were progressively introduced in most developed countries to replace the very efficient but more reactogenic whole cell vaccine (wP)^5–9^. This trend is continuing as a growing number of less privileged countries adopt aP vaccines. Unfortunately, this switch led to a decrease in vaccine efficacy, as immunity induced by aP vaccines wanes rapidly such that repeated boosters are required to maintain protection^10–15^.

Most industrialized countries have recommended the implementation of aP boosters beyond infancy and childhood, but their impact is insufficient: additional measures are needed especially during the cyclic outbreaks of *B. Pertussis*, as currently reported from many countries. One puzzling observation is that pertussis occurs in fully vaccinated individuals, despite a long (1 to 3 weeks) incubation period which should be sufficient to recall memory cells and interrupt bacterial proliferation^16^. Moreover, aP vaccines were designed to limit severe infant pertussis and do not prevent colonization and therefore cannot hamper transmission/carriage^17–19^. Therefore, vaccinated individuals participate in transmission chains, precluding disease control.

The study of immune responses following pertussis challenge has been best achieved in non-human-primates (NHPs)^18,20,21^. They are the only available animal models to allow studying pertussis transmission; however, to our knowledge no study showed that pertussis antibodies wane in NHPs as rapidly as in humans. This prevents the analysis of immune memory reactivation in the absence of circulating serum antibodies^22^. To overcome this issue, we have previously developed an adoptive transfer murine model, enabling the elimination of circulating antibodies, and thus better mimicking the human situation^23^.

Here, we used this adapted murine model to assess whether - and under which conditions - a post-exposure aP vaccine booster could recall vaccine-induced memory and accelerate bacterial clearance both in the lungs and the trachea.

## Materials and methods

### Mice

Adult female BALB/cByJ mice were purchased from Charles River (L’Arbresle, France), kept under specific pathogen free conditions and used in all experiments. Mice were used at 6–8 weeks of age. Mice were euthanized by carbon dioxide (CO₂) overdose, delivered in a gradually filled chamber.

- Immunizations.

Mice were immunized intra-muscularly (i.m.) on days 0 and 21 (priming) and/or on day 1 or 3 post-*B. pertussis* exposure (boosting) with 1/5th of a human dose (50 µl in both hind legs) of DTaP-IPV (Infanrix^®^, GlaxoSmithKline). Some mice (Figure S1) were boosted on day 1 post-*B. pertussis* exposure with 50 µl in both hind legs of Tdap (Boostrix^®^, GlaxoSmithKline, 1/5th of the human dose). Passive immunization (Figure S2) was performed on day 5 after *B. pertussis* exposure by administering intra-peritoneally (i.p.) 200 µl of hyperimmune sera (collected on day 41 after two DTaP-IPV doses given in naïve mice at days 0 and 21).

- Adoptive transfer.

Spleens were harvested 42 days after DTaP-IPV priming and boosting. Single cell suspensions were obtained by mechanical disruption and processed for red blood cell lysis. 25 × 10^6^ splenocytes (experimentally defined as optimizing the recall of immune memory, unpublished data) were resuspended in 100 µl of saline solution and transferred intravenously (i.v.) by retro-orbital injection into naïve mice.

- Bordetella pertussis challenge.

Streptomycin-resistant *Bordetella pertussis* 18,323 (U.S. Food and Drug Administration) were grown on Bordet-Gengou agar (Difco) supplemented with 1% glycerol, 10% defibrinated sheep blood (Chemie Brunschwig AG) and 100 µg/ml streptomycin. 1 × 10^6^ colony-forming units (CFU) were instilled intranasally in a volume of 20 µL into mice anesthetized by i.p. injection of Ketasol^®^ (100 mg/kg; Graeub) and Rompun^®^ (10 mg/kg; Bayer). Mice were sacrificed 2–3 h after infection for quantification of the initial numbers of viable *B. pertussis* CFUs in the lungs and trachea, and at different time-points post challenge for determination of bacterial colonization. Lungs and trachea homogenates were plated onto Bordet-Gengou agar plates supplemented with 1% glycerol, 10% defibrinated sheep blood (Chemie Brunschwig AG) and 100 µg/ml streptomycin; and the number of CFUs was counted after 4 days of incubation at 37 °C.

- Antibodies quantification.

*B. pertussis* antigen-specific antibody titers were determined by ELISAs using 96-well plates (Nunc MaxiSorp™; ThermoFischer Scientific) coated with pertussis toxin (PT, WHO International Standard *B- pertussis* Toxin 2nd IS, NIBSC code: 15/126) (1 µg/ml) or filamentous hemagglutinin (FHA, The Native Antigen Company) (1 µg/ml). Wells were incubated with 2-fold serial dilutions of individual or pooled mouse prior to incubation with secondary horseradish peroxidase (HRP) conjugated anti-mouse IgG (Invitrogen). The optical density of each well was measured at 405 nm and the data analysed with SoftMax Pro software. IgG titers were expressed as Log10 in reference to a pool of hyperimmune sera harvested from vaccinated mice to quantify antibody levels with precision and minimal inter-plate variation^24^. Titers under 10^2^ are not detected by the assay.

### Statistical analysis

Values are expressed as mean ± SEM. Statistical analysis were performed using one-way ANOVA followed by a Tukey multiple comparison test. All analyses were done using the GraphPad Prism version 10 (GraphPad Software, San Diego, CA, USA).

## Results

### Post-exposure boosting leads to rapid and strong antibody recall

**Fig. 1:**
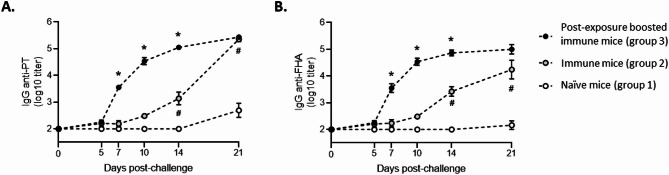
Post-exposure boosting enables a rapid and strong antibody recall. Splenocytes of DTaP-IPV-vaccinated mice were transferred into naïve recipient mice (“immune mice”, group 2 and 3), but not into controls (“naïve mice”). All mice were challenged with *B. pertussis* a week later. In group 3, a post-exposure DTaP-IPV boost was administered one day after challenge. PT-specific IgG (A) and FHA-specific IgG (B) antibody titers were determined by ELISA on sera collected on the day of challenge (day 0) and at days 5, 7, 10, 14 and 21 post challenge. Data represents mean +/- SEM from 2 different experiments, including between 4 to 9 mice per group depending on the time point. * *p* < 0.0001 comparing group 2 and 3, i.e. with and without post-exposure boosting; ♯ *p* < 0.0001 comparing group 1 and 2, i.e. in previously immunized versus naïve mice.

**Fig. 2:**
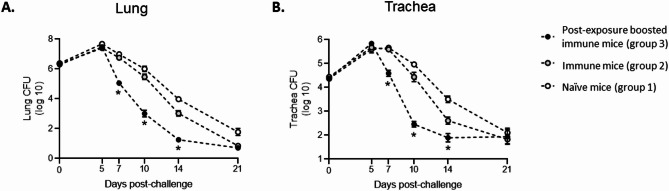
Post-exposure boosting accelerates bacterial clearance both in the lungs and the trachea. Splenocytes of DTaP-IPV-vaccinated mice were transferred into naïve recipient mice which were challenged with *B. pertussis* a week later (group 2 and 3). DTaP-IPV boosting was administered one day after challenge in mice of group 3. CFUs were counted in the lungs (**A**) and the trachea (**B**) at 3 h and at days 5, 7, 10, 14 and 21 post *B. pertussis* challenge, and are displayed on a logarithmic scale. Data represents mean +/- SEM from 5 different experiments, and between 7 to 24 mice per group depending on the time point. * *p* < 0.01 comparing group 2 and 3, i.e. with and without post-exposure aP boosting.

In naïve control mice (group 1), serum antibodies to PT only started to increase 3 weeks (day 21) after *B. pertussis* challenge (Fig. 1A), with no increase in anti-FHA antibodies. In recipients of splenocytes from immunized mice (i.e. “immune mice”), PT and FHA antibodies were undetectable on the day of *B. pertussis* challenge, confirming the absence of circulating serum antibodies following the adoptive transfer of immune splenocytes. After *B. pertussis* challenge, these antibodies increased significantly faster in immune (group 2) compared to naïve control mice. It nevertheless still required 2 weeks (day 14) for PT- and FHA-specific-IgG to reach statistically significant (10^3^) and 3 weeks (day 21) to reach high (10^4–5^) ELISA titers. This pattern is similar to the 3-weeks delay required for the serological diagnosis of pertussis in humans^25^. In contrast, boosting immune mice with DTaP-IPV one day after *B. pertussis* challenge (group 3) rapidly recalled both PT- and FHA-specific-IgG antibodies which reached significantly higher levels on days 7, 10 and 14 in boosted compared to non-boosted immune mice (group 3 vs. group 2, Fig. 1). These responses were similar to those observed in another set of experiments in which adoptively transferred recipients (“immune mice”) were boosted in the absence of *B. pertussis* challenge (Fig. 2).

- Post-exposure boosting accelerates *Bordetella pertussis* clearance in the lungs and trachea.

We assessed whether the faster and stronger increase of *B. pertussis*-specific antibodies observed in immune mice boosted with DTaP-IPV one day after *B. pertussis* exposure impacted lung and tracheal bacterial loads. *B. pertussis* actively replicated in naïve mice and in recipients of immune splenocytes, reaching higher numbers of CFU in the lungs (Fig. 2A) and trachea (Fig. 2B) on day 5 than day 0, and slowly declining during the following 2 weeks. Post-exposure DTaP-IPV boosting significantly accelerated bacterial clearance in both the lungs and trachea, which were cleared of *B. pertussis* 2 weeks post challenge, as illustrated by the significantly lower numbers of CFUs on day 7, 10 and 14 (Fig. 2).

Delayed post-exposure boosting of recipients of immune splenocytes remained effective: lung and tracheal bacterial clearance was still faster when aP boosting was performed on day 3 after exposure than in non-boosted mice (Figure S3). Boosting on day 3 compared to day 1 after *B. pertussis* exposure only transiently delayed clearance, with a significant difference only observed on day 7 but no more on days 10, 14 and 21 after challenge.

Last, *B. pertussis* immunity could be recalled as efficiently with a lower vaccine dose. Day 1 post-exposure boosting with Tdap, which contains 3 times less PT and FHA than DTaP-IPV, was similarly effective on *B. pertussis* clearance from lungs and trachea (Figure S1).

- Post-exposure active and passive immunization similarly reduce bacterial clearance.

**Fig. 3:**
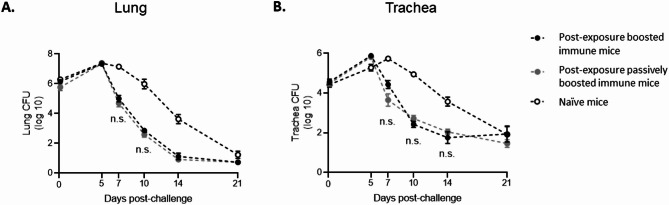
Post-exposure active and passive immunizations exert similar influences on bacterial clearance. Splenocytes of DTaP-IPV-vaccinated mice were transferred into naïve recipient mice which were challenged a week later with *B. pertussis*. The group “naïve mice” did not receive any transferred splenocytes but was challenged with *B. pertussis* at the same time as the other groups. One group received a post-exposure DTaP-IPV booster i.m. one day after challenge while the second group received immune sera i.p. 5 days after challenge. CFUs were counted in the lungs (**A**) and the trachea (**B**) at 3 h and days 5, 7, 10, 14 and 21 post *B. pertussis* challenge, and are displayed on a logarithmic scale. Data represents mean +/- SEM from 2 different experiments, 10 mice per group. n. s.: *p* > 0.05 comparing post-exposure active and passive immunization.

The correlation between a faster/stronger increase of antibodies elicited by post-exposure boosting with a faster bacterial clearance in both the trachea and the lungs suggests that antibodies are the main effectors – without however excluding the requirement of T cell help. This was assessed by the post-exposure injection of immune sera from DTaP-IPV vaccinated mice into recipients of immune splenocytes. To best mimic the time needed for memory B cells to differentiate into antibody-secreting cells, passive immunization was provided on day 5 after *B. pertussis* challenge. Post-exposure passive immunization accelerated bacterial clearance in lungs and trachea as efficiently as post-exposure DTaP-IPV (Fig. 3A and B). Thus, providing PT/FHA-specific antibodies was sufficient to accelerate bacterial clearance. The kinetics of PT/FHA-specific serum antibodies were similar with passive or active post-exposure immunization (Figure S2).

## Discussion

The current aP vaccination schedule remains suboptimal, as its transient efficacy fails to prevent carriage and transmissibility and thus does not efficiently reduce disease prevalence and contamination of high-risk neonates or infants too young to be vaccinated^14^. Using a specifically developed murine model^23^, we show that *B. pertussis* exposure alone leads to a slow and weak reactivation of vaccine-induced immune memory, resulting in prolonged respiratory bacterial carriage, whereas post-exposure aP booster vaccination induces a prompt and robust immune recall.

Infection with *B. pertussis* is very slow at recalling vaccine-induced memory B cells and triggering antibody responses in humans^16,26^. We show that this delayed immune reactivation is similar in our adoptive murine model and that it is associated with prolonged bacterial loads in the lungs and trachea (Figs. 1 and 2). This slow and weak reactivation of vaccine-induced immune memory upon *B. pertussis* infection could result from a combination of factors. First, the incubation period of *B. pertussis* is long, delaying memory B cell reactivation^27^. Second, memory B cell reactivation requires a sufficient amount of antigen to trigger plasma cell differentiation. Since *B. pertussis* is a strictly mucosal pathogen, without bacteraemia except in severely immune-compromised individuals^28^, we postulate that there is limited antigen dissemination to the draining lymph nodes where memory B cells essentially reside. Last, *B. pertussis* exerts local immunomodulatory effects which limit the activation and migration of (antigen-loaded) dendritic cells to the draining lymph nodes^29,30^. Thus, the rapid reactivation of pertussis immunity does not occur after *B. pertussis* exposure, resulting in recurrent infection and symptoms^31^.

From a clinical perspective, this delayed immune reactivation is likely to translate into prolonged respiratory bacterial carriage, thereby sustaining transmission to susceptible individuals, including young infants, even in highly vaccinated populations.

In contrast, giving an aP vaccine even a few days after *B. pertussis* exposure induces a faster and stronger increase of anti-PT and anti-FHA serum IgG (mucosal antibody responses were not tested), reflecting a more effective reactivation of vaccine-induced memory B cells. This post-exposure boosting accelerates *B. pertussis* clearance, with similar patterns and kinetics as the post-exposure administration of immune serum (passive immunization). This strongly suggests a predominant role of memory B cell reactivation without excluding a contribution of T cells (additional to their B cell helper effect). Further experiments could characterize the populations of memory cells required for protection. Indeed, although correlates of protection are not well defined for pertussis^32^, a critical role has been attributed to antibodies and to mucosal CD4^+^ Th1/Th17 cells in long-lasting protection and prevention of asymptomatic carriage^33,34^. A recent controlled immune infection studies with *B. pertussis* reports that higher humoral (i.e. serum IgG and IgA specific antibodies) and CD4^+^ T-cell responses are associated with protection against colonisation^35^.Th2-prone female BALB/c mice were used exclusively, whereas male mice or C57BL/6 mice with more Th1-skewed responses were not evaluated. Whether this would change the impact of post-exposure boosting - and whether a given strain is more relevant to the human situation or not - remains unknown. This is not addressed in this study where we only used the low pathogenicity *B.pertussis* strain 18,232^36^, adapting its challenge dose for sufficient and reproductive colonisation and replication in the lungs and trachea.

Importantly, post-exposure boosting does not only increase antibody responses but significantly reduces bacterial load in the lungs and in the trachea. To what extent this would reduce transmissibility cannot be studied in murine models, since mice remain asymptomatic. Interrupting transmission is however key in disease control strategies against pertussis, a highly transmissible infection that spreads from case to close contact via respiratory droplets^19^. To our knowledge, none of the currently licensed aP vaccines have been reported to prevent or reduce *B. pertussis* carriage, and this was not tested in this study.

Currently *B. pertussis* infection is classically managed with antibiotics administered during the catarrhal phase, which may reduce contagiousness but do not significantly alter the course of disease once paroxysmal symptoms are established, and infected individuals may continue to transmit the infection for approximately 3 weeks^37^. In this context, a post-exposure vaccination strategy reducing the duration of infection could represent a complementary and potentially more effective approach, both in terms of antibiotic sparing and epidemic control. In our preclinical model, post-exposure administration of acellular pertussis vaccines rapidly reactivated vaccine-induced immunity and substantially reduced respiratory bacterial burden, with effects comparable to passive immunization. In the controlled human infection model with *B. pertussis*^38^ higher pre-inoculation serum IgG/IgA antibody concentrations to Bp antigens and specific CD4^+^ T cell responses correlated with less colonization after challenge. Testing the effect of early post-exposure booster vaccination on colonization and analysing results by the stratification of preexisting serum Bp-specific antibodies would be an elegant way to assess the impact of post-exposure boosting in humans, and to define how well some adapted murine model may mimic the human situation. If confirmed in controlled infection human challenge models or clinical studies, early post-exposure vaccination of contacts could help limit transmission within households and paediatric settings, thereby improving the protection of young infants at highest risk of severe disease.

## Supplementary Information

Below is the link to the electronic supplementary material.

Supplementary Material 1

Supplementary Material 2

Supplementary Material 3

## Data Availability

The data that support the findings of this study are available from the corresponding author upon reasonable request.
