# Supplementary figures and images for: Post-exposure booster vaccination recalls vaccine-induced memory and accelerates Bordetella pertussis clearance in murine lungs and trachea
Source: Sci Rep. 2026 Jul 20;16:22680. doi: 10.1038/s41598-026-56963-y (PMC13385624; doi:10.1038/s41598-026-56963-y)

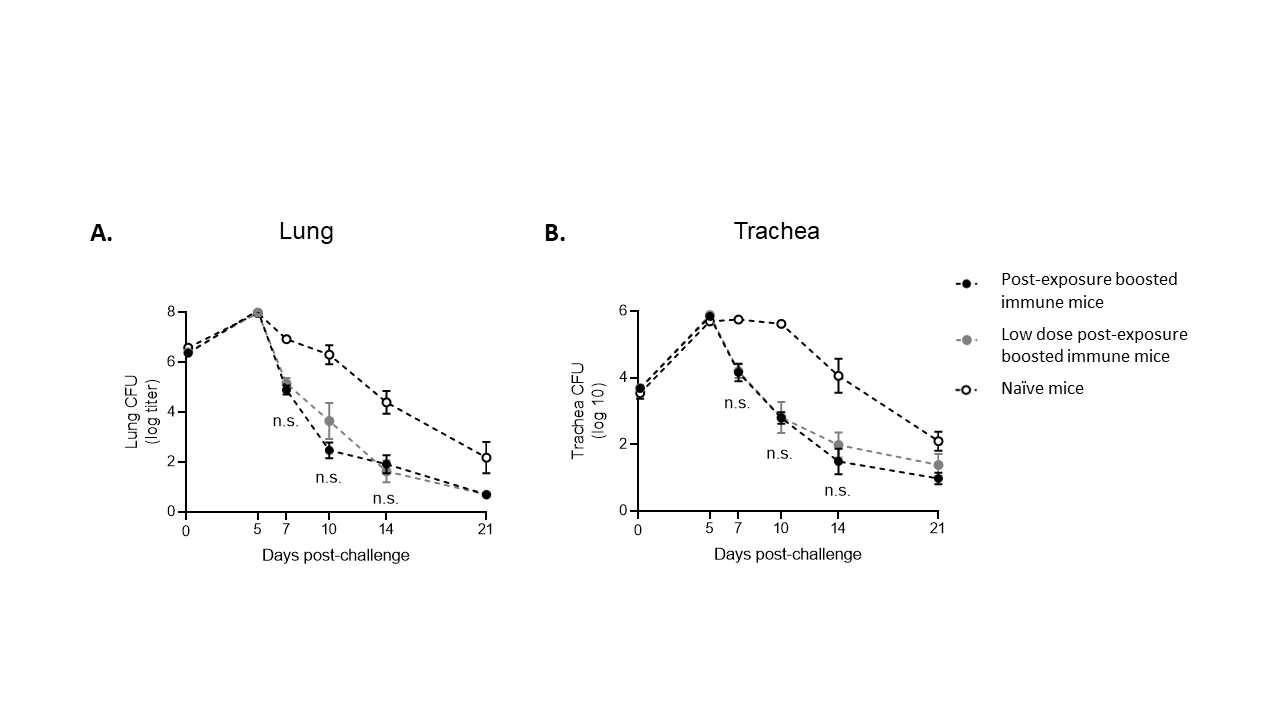

Supplement: Supplementary file 1 — Supplementary Material 1 [file 41598_2026_56963_MOESM1_ESM.tif]

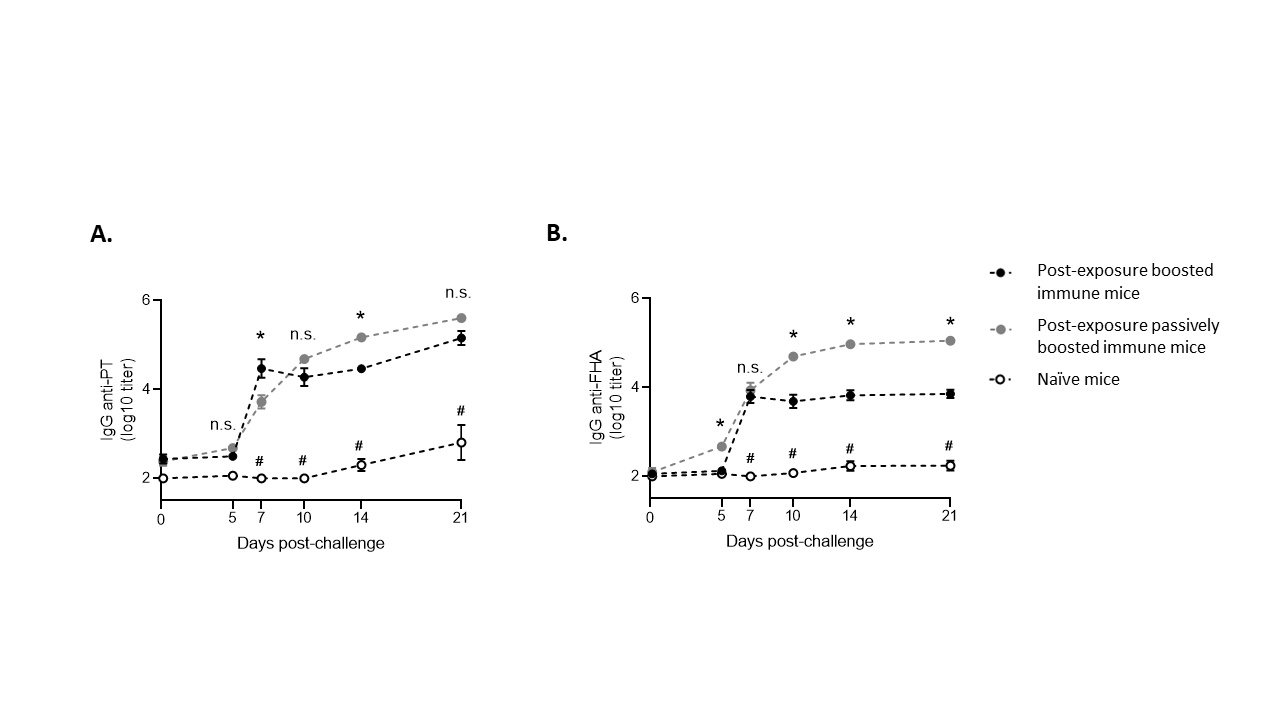

Supplement: Supplementary file 2 — Supplementary Material 2 [file 41598_2026_56963_MOESM2_ESM.tif]

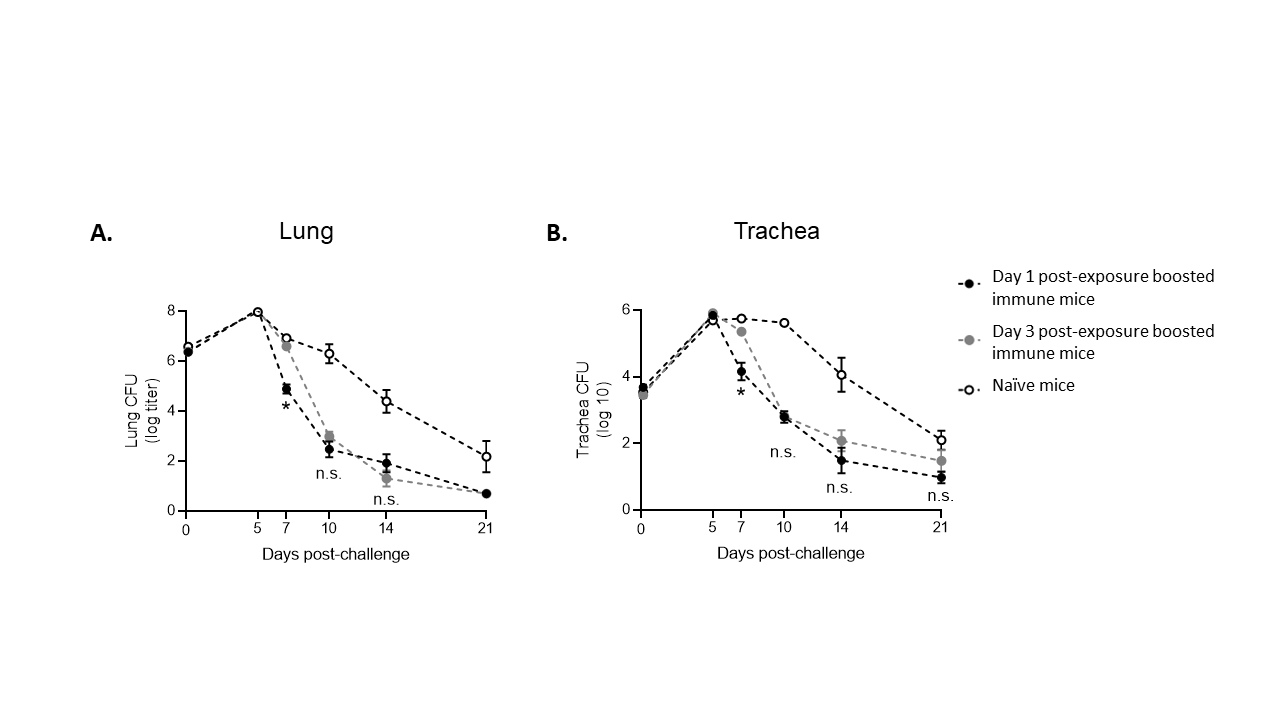

Supplement: Supplementary file 3 — Supplementary Material 3 [file 41598_2026_56963_MOESM3_ESM.tif]
